# (2,6-Difluoro­benzophenone)tris­(trimethyl­phosphine)cobalt(0)

**DOI:** 10.1107/S1600536809012367

**Published:** 2009-04-08

**Authors:** Jun Ding, Xiao-Yan Li

**Affiliations:** a School of Chemistry and Chemical Engineering, Shandong University, Jinan 250100, People’s Republic of China

## Abstract

In the title compound, [Co(C_13_H_8_F_2_O)(C_3_H_9_P)_3_], the cobalt(0) atom is coordinated by three trimethyl­phosphine ligands and a π-coordinated carbonyl group of the 2,6-difluoro­benzo­phenone ligand in a distorted tetra­hedral geometry. The Co—O and Co—C distances are 1.896 (2) and 2.049 (4) Å, respectively.

## Related literature

For general background to the activation of C—F bonds in organometallic chemistry and catalyst development, see: Kiplinger *et al.* (1994 ▶); Saunders (1996 ▶); Li *et al.* (2006 ▶).
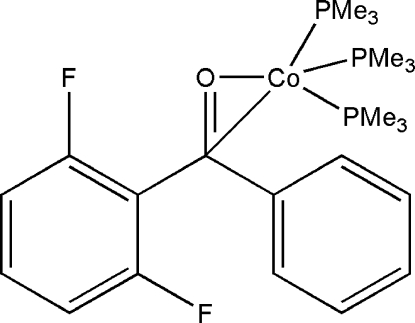


## Experimental

### 

#### Crystal data

[Co(C_13_H_8_F_2_O)(C_3_H_9_P)_3_]
*M* *_r_* = 505.34
Monoclinic, 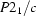

*a* = 14.214 (5) Å
*b* = 9.820 (4) Å
*c* = 19.241 (7) Å
β = 101.773 (6)°
*V* = 2629.3 (16) Å^3^
*Z* = 4
Mo *K*α radiation
μ = 0.86 mm^−1^
*T* = 273 K
0.32 × 0.27 × 0.26 mm

#### Data collection

Bruker APEXII CCD diffractometer
Absorption correction: multi-scan (*SADABS*; Sheldrick, 1996 ▶) *T*_min_ = 0.759, *T*_max_ = 0.800
13574 measured reflections
5065 independent reflections
3278 reflections with *I* > 2σ(*I*)
*R*_int_ = 0.047

#### Refinement

*R*[*F* ^2^ > 2σ(*F* ^2^)] = 0.044
*wR*(*F* ^2^) = 0.128
*S* = 0.99
5065 reflections
262 parameters
H-atom parameters constrained
Δρ_max_ = 0.38 e Å^−3^
Δρ_min_ = −0.31 e Å^−3^

### 

Data collection: *APEX2* (Bruker, 2005 ▶); cell refinement: *SAINT* (Bruker, 2005 ▶); data reduction: *SAINT*; program(s) used to solve structure: *SHELXS97* (Sheldrick, 2008 ▶); program(s) used to refine structure: *SHELXL97* (Sheldrick, 2008 ▶); molecular graphics: *ORTEP-3 for Windows* (Farrugia, 1997 ▶); software used to prepare material for publication: *WinGX* (Farrugia, 1999 ▶).

## Supplementary Material

Crystal structure: contains datablocks I, global. DOI: 10.1107/S1600536809012367/xu2497sup1.cif

Structure factors: contains datablocks I. DOI: 10.1107/S1600536809012367/xu2497Isup2.hkl

Additional supplementary materials:  crystallographic information; 3D view; checkCIF report

## Figures and Tables

**Table 1:** Selected bond lengths (Å)

Co1—P2	2.2304 (11)
Co1—P3	2.2081 (12)
Co1—P4	2.2632 (13)

**Fig. 1.**
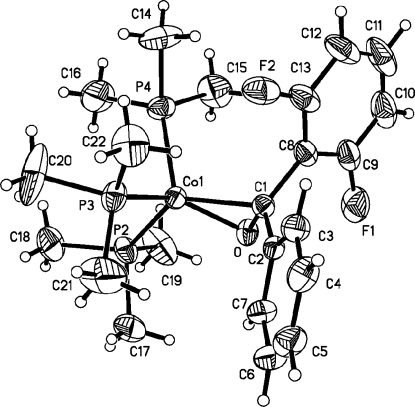
The molecular structure of the title compound, displacement ellipsoids are drawn at the 25% probability level.

**Fig. 2.**
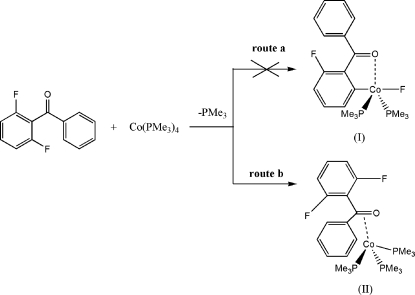
The formation of the title compound.

## supplementary crystallographic information

### Comment

The activation of carbon-fluorine bonds is of great importance in organometallic chemistry and catalyst development because this type of reaction contributes tothe fundamental understanding of reactivity of stable bonds and selective replacement of F atoms (Kiplinger *et al.*, 1994; Saunders, 1996). Recently we have reported cyclometalation reactions involving C—F bond activation at a cobalt(I) center with azine as an anchoring group, which afforded the first complex containing a C—Co—F fragment (Li *et al.*, 2006).

We tried to synthesis compound I (Scheme 2) according to the route a in Fig. 2 throught the reaction of 2,6-difluorobenzophenone with tetra(trimethylphosphine)cobalt(0), but the reaction was actually occurred by route b (Scheme 2). Compound II was isolated as red crystals and its molecular structure is shown in Fig. 1. The cobalt atom is π-coordinated with carbonyl group. The Co—O and Co—C bond lengths are 1.896 (2) Å and 2.049 (4) Å (Table 1).

### Experimental

The title compound was synthesized from the reaction of tetra(trimethyl phosphine)cobalt (1.2 g, 3.3 mmol) and 2,6-difluorobenzophenone (0.72 g, 3.3 mmol) in pentane (40 ml) for 24 h at 298 K. After filtration, the title complex was obtained from filtrate as red crystals at 246 K.

### Refinement

The H atoms were geometrically placed and treated as riding atoms with C—H = 0.96 Å and *U*_iso_(H)=1.5U_eq_(C) for methyl H atoms, C—H = 0.93 Å and *U*_iso_(H)=1.2U_eq_(C) for arometic H atoms.

### Crystal data

**Table:**

[Co(C_13_H_8_F_2_O)(C_3_H_9_P)_3_]	*F*(000) = 1060
*M**_r_* = 505.34	*D*_x_ = 1.277 Mg m^−^^3^
Monoclinic, *P*2_1_/*c*	Mo *K*α radiation, λ = 0.71073 Å
Hall symbol: -P 2ybc	Cell parameters from 2218 reflections
*a* = 14.214 (5) Å	θ = 2.3–22.0°
*b* = 9.820 (4) Å	µ = 0.86 mm^−^^1^
*c* = 19.241 (7) Å	*T* = 273 K
β = 101.773 (6)°	Prism, red
*V* = 2629.3 (16) Å^3^	0.32 × 0.27 × 0.26 mm
*Z* = 4	

### Data collection

**Table:**

Bruker APEX2 CCD diffractometer	5065 independent reflections
Radiation source: fine-focus sealed tube	3278 reflections with *I* > 2σ(*I*)
graphite	*R*_int_ = 0.047
φ and ω scans	θ_max_ = 25.9°, θ_min_ = 1.5°
Absorption correction: multi-scan (*SADABS*; Sheldrick, 1996)	*h* = −17→13
*T*_min_ = 0.759, *T*_max_ = 0.800	*k* = −12→10
13574 measured reflections	*l* = −21→23

### Refinement

**Table:**

Refinement on *F*^2^	Secondary atom site location: difference Fourier map
Least-squares matrix: full	Hydrogen site location: inferred from neighbouring sites
*R*[*F*^2^ > 2σ(*F*^2^)] = 0.044	H-atom parameters constrained
*wR*(*F*^2^) = 0.128	*w* = 1/[σ^2^(*F*_o_^2^) + (0.0646*P*)^2^] where *P* = (*F*_o_^2^ + 2*F*_c_^2^)/3
*S* = 0.99	(Δ/σ)_max_ < 0.001
5065 reflections	Δρ_max_ = 0.38 e Å^−^^3^
262 parameters	Δρ_min_ = −0.31 e Å^−^^3^
0 restraints	Extinction correction: *SHELXL*
Primary atom site location: structure-invariant direct methods	Extinction coefficient: 0.0037 (5)

### Special details

**Table:**

Geometry. All e.s.d.'s (except the e.s.d. in the dihedral angle between two l.s. planes) are estimated using the full covariance matrix. The cell e.s.d.'s are taken into account individually in the estimation of e.s.d.'s in distances, angles and torsion angles; correlations between e.s.d.'s in cell parameters are only used when they are defined by crystal symmetry. An approximate (isotropic) treatment of cell e.s.d.'s is used for estimating e.s.d.'s involving l.s. planes.
Refinement. Refinement of *F*^2^ against ALL reflections. The weighted *R*-factor *wR* and goodness of fit *S* are based on *F*^2^, conventional *R*-factors *R* are based on *F*, with *F* set to zero for negative *F*^2^. The threshold expression of *F*^2^ > σ(*F*^2^) is used only for calculating *R*-factors(gt) *etc*. and is not relevant to the choice of reflections for refinement. *R*-factors based on *F*^2^ are statistically about twice as large as those based on *F*, and *R*- factors based on ALL data will be even larger.

### Fractional atomic coordinates and isotropic or equivalent isotropic displacement parameters (Å^2^)

**Table:**

	*x*	*y*	*z*	*U*_iso_*/*U*_eq_	
C22	0.0242 (3)	0.2517 (5)	0.0761 (3)	0.0976 (17)	
H22A	0.0310	0.3485	0.0723	0.146*	
H22B	−0.0280	0.2321	0.0993	0.146*	
H22C	0.0112	0.2120	0.0295	0.146*	
C21	0.1219 (4)	0.2248 (7)	0.2183 (3)	0.146 (3)	
H21A	0.1384	0.3188	0.2275	0.219*	
H21B	0.1636	0.1686	0.2521	0.219*	
H21C	0.0565	0.2102	0.2225	0.219*	
C20	0.1004 (3)	0.0027 (5)	0.1249 (4)	0.156 (3)	
H20A	0.1527	−0.0510	0.1503	0.234*	
H20B	0.0843	−0.0270	0.0764	0.234*	
H20C	0.0456	−0.0079	0.1464	0.234*	
Co1	0.27686 (3)	0.23243 (4)	0.10451 (2)	0.04168 (16)	
P2	0.37675 (7)	0.09320 (11)	0.17586 (5)	0.0577 (3)	
P3	0.13547 (7)	0.18019 (11)	0.12795 (6)	0.0611 (3)	
P4	0.27110 (8)	0.12824 (11)	−0.00119 (5)	0.0638 (3)	
C2	0.2322 (2)	0.5172 (3)	0.14070 (17)	0.0433 (8)	
C1	0.2824 (2)	0.4393 (3)	0.09203 (17)	0.0451 (8)	
C8	0.2790 (3)	0.4990 (4)	0.01902 (19)	0.0540 (9)	
C3	0.1474 (3)	0.5910 (4)	0.1188 (2)	0.0617 (10)	
H3	0.1196	0.5968	0.0708	0.074*	
C7	0.2722 (3)	0.5159 (4)	0.21376 (19)	0.0603 (10)	
H7	0.3286	0.4676	0.2302	0.072*	
C17	0.4027 (3)	0.1515 (5)	0.2682 (2)	0.0974 (16)	
H17A	0.3443	0.1529	0.2860	0.146*	
H17B	0.4295	0.2415	0.2705	0.146*	
H17C	0.4478	0.0906	0.2964	0.146*	
C6	0.2294 (3)	0.5848 (5)	0.2616 (2)	0.0764 (12)	
H6	0.2588	0.5849	0.3094	0.092*	
C13	0.1996 (4)	0.4964 (4)	−0.0364 (2)	0.0715 (12)	
C4	0.1040 (3)	0.6561 (4)	0.1688 (2)	0.0706 (12)	
H4	0.0465	0.7026	0.1534	0.085*	
C12	0.2003 (5)	0.5465 (5)	−0.1037 (2)	0.0985 (18)	
H12	0.1452	0.5432	−0.1393	0.118*	
C5	0.1444 (3)	0.6530 (5)	0.2400 (2)	0.0744 (12)	
H5	0.1147	0.6962	0.2728	0.089*	
C14	0.1648 (4)	0.1393 (6)	−0.0739 (2)	0.1102 (19)	
H14A	0.1436	0.2322	−0.0798	0.165*	
H14B	0.1141	0.0840	−0.0628	0.165*	
H14C	0.1811	0.1075	−0.1172	0.165*	
C19	0.4970 (3)	0.1005 (6)	0.1562 (3)	0.115 (2)	
H19A	0.4943	0.0720	0.1081	0.172*	
H19B	0.5392	0.0412	0.1879	0.172*	
H19C	0.5208	0.1922	0.1622	0.172*	
C9	0.3600 (4)	0.5615 (4)	0.0023 (2)	0.0722 (12)	
C15	0.3646 (4)	0.1877 (6)	−0.0472 (3)	0.1090 (18)	
H15A	0.3619	0.2852	−0.0511	0.163*	
H15B	0.3542	0.1483	−0.0938	0.163*	
H15C	0.4265	0.1608	−0.0208	0.163*	
C18	0.3626 (4)	−0.0900 (5)	0.1899 (3)	0.0966 (16)	
H18A	0.3488	−0.1356	0.1448	0.145*	
H18B	0.3106	−0.1044	0.2140	0.145*	
H18C	0.4209	−0.1257	0.2181	0.145*	
C16	0.2929 (5)	−0.0555 (5)	−0.0030 (3)	0.122 (2)	
H16A	0.3485	−0.0783	0.0326	0.184*	
H16B	0.3037	−0.0810	−0.0489	0.184*	
H16C	0.2380	−0.1036	0.0063	0.184*	
C10	0.3645 (5)	0.6093 (5)	−0.0633 (3)	0.1023 (19)	
H10	0.4212	0.6469	−0.0718	0.123*	
C11	0.2840 (6)	0.6012 (6)	−0.1169 (3)	0.115 (3)	
H11	0.2862	0.6329	−0.1621	0.139*	
F2	0.11615 (18)	0.4390 (3)	−0.02486 (12)	0.0844 (7)	
F1	0.4389 (2)	0.5788 (3)	0.05420 (16)	0.0992 (9)	
O	0.36561 (15)	0.3788 (2)	0.12166 (12)	0.0507 (6)	

### Atomic displacement parameters (Å^2^)

**Table:**

	*U*^11^	*U*^22^	*U*^33^	*U*^12^	*U*^13^	*U*^23^
C22	0.049 (3)	0.106 (4)	0.136 (4)	0.015 (2)	0.015 (3)	0.016 (3)
C21	0.092 (4)	0.265 (9)	0.095 (4)	−0.069 (5)	0.055 (3)	−0.020 (4)
C20	0.066 (3)	0.063 (4)	0.336 (9)	−0.020 (3)	0.033 (4)	0.019 (5)
Co1	0.0387 (3)	0.0395 (3)	0.0451 (3)	0.00219 (19)	0.00419 (19)	0.0017 (2)
P2	0.0495 (6)	0.0589 (7)	0.0628 (6)	0.0071 (5)	0.0071 (5)	0.0193 (5)
P3	0.0434 (5)	0.0559 (7)	0.0824 (7)	−0.0070 (4)	0.0089 (5)	0.0030 (5)
P4	0.0753 (7)	0.0568 (7)	0.0559 (6)	0.0111 (5)	0.0058 (5)	−0.0120 (5)
C2	0.0445 (19)	0.039 (2)	0.0469 (19)	−0.0049 (15)	0.0095 (16)	−0.0005 (15)
C1	0.0457 (19)	0.043 (2)	0.0458 (19)	0.0017 (15)	0.0072 (16)	0.0023 (16)
C8	0.074 (3)	0.041 (2)	0.051 (2)	0.0144 (18)	0.024 (2)	0.0037 (17)
C3	0.072 (3)	0.054 (3)	0.057 (2)	0.010 (2)	0.010 (2)	−0.0017 (19)
C7	0.060 (2)	0.066 (3)	0.051 (2)	−0.0006 (19)	0.0030 (19)	−0.0088 (19)
C17	0.106 (4)	0.106 (4)	0.066 (3)	−0.004 (3)	−0.015 (3)	0.022 (3)
C6	0.094 (3)	0.082 (3)	0.053 (2)	−0.008 (3)	0.014 (2)	−0.017 (2)
C13	0.101 (3)	0.064 (3)	0.051 (3)	0.037 (3)	0.019 (3)	0.009 (2)
C4	0.064 (3)	0.058 (3)	0.093 (3)	0.009 (2)	0.021 (2)	−0.010 (2)
C12	0.157 (5)	0.085 (4)	0.051 (3)	0.058 (4)	0.016 (3)	0.014 (3)
C5	0.083 (3)	0.077 (3)	0.074 (3)	−0.008 (3)	0.039 (3)	−0.022 (2)
C14	0.109 (4)	0.129 (5)	0.074 (3)	0.024 (3)	−0.025 (3)	−0.042 (3)
C19	0.050 (3)	0.169 (6)	0.127 (4)	0.028 (3)	0.020 (3)	0.073 (4)
C9	0.105 (4)	0.051 (3)	0.072 (3)	0.006 (2)	0.046 (3)	0.007 (2)
C15	0.140 (5)	0.108 (5)	0.093 (4)	0.018 (4)	0.059 (4)	−0.012 (3)
C18	0.116 (4)	0.065 (3)	0.106 (4)	0.022 (3)	0.017 (3)	0.032 (3)
C16	0.201 (6)	0.060 (4)	0.101 (4)	0.021 (4)	0.017 (4)	−0.020 (3)
C10	0.167 (6)	0.066 (3)	0.097 (4)	0.006 (3)	0.081 (4)	0.012 (3)
C11	0.222 (8)	0.072 (4)	0.077 (4)	0.053 (4)	0.088 (5)	0.025 (3)
F2	0.0767 (16)	0.101 (2)	0.0655 (15)	0.0232 (15)	−0.0084 (13)	0.0003 (13)
F1	0.099 (2)	0.092 (2)	0.116 (2)	−0.0297 (16)	0.0463 (19)	0.0059 (17)
O	0.0400 (13)	0.0444 (14)	0.0660 (15)	−0.0008 (10)	0.0070 (11)	0.0052 (11)

### Geometric parameters (Å, °)

**Table:**

C22—P3	1.829 (4)	C7—H7	0.9300
C22—H22A	0.9600	C17—H17A	0.9600
C22—H22B	0.9600	C17—H17B	0.9600
C22—H22C	0.9600	C17—H17C	0.9600
C21—P3	1.841 (5)	C6—C5	1.370 (6)
C21—H21A	0.9600	C6—H6	0.9300
C21—H21B	0.9600	C13—F2	1.372 (5)
C21—H21C	0.9600	C13—C12	1.386 (6)
C20—P3	1.810 (5)	C4—C5	1.374 (6)
C20—H20A	0.9600	C4—H4	0.9300
C20—H20B	0.9600	C12—C11	1.376 (7)
C20—H20C	0.9600	C12—H12	0.9300
Co1—O	1.896 (2)	C5—H5	0.9300
Co1—C1	2.049 (4)	C14—H14A	0.9600
Co1—P2	2.2304 (11)	C14—H14B	0.9600
Co1—P3	2.2081 (12)	C14—H14C	0.9600
Co1—P4	2.2632 (13)	C19—H19A	0.9600
P2—C19	1.826 (4)	C19—H19B	0.9600
P2—C17	1.830 (4)	C19—H19C	0.9600
P2—C18	1.836 (5)	C9—F1	1.351 (5)
P4—C16	1.833 (5)	C9—C10	1.361 (6)
P4—C15	1.835 (5)	C15—H15A	0.9600
P4—C14	1.842 (4)	C15—H15B	0.9600
C2—C3	1.395 (5)	C15—H15C	0.9600
C2—C7	1.405 (5)	C18—H18A	0.9600
C2—C1	1.498 (4)	C18—H18B	0.9600
C1—O	1.343 (4)	C18—H18C	0.9600
C1—C8	1.514 (5)	C16—H16A	0.9600
C8—C13	1.386 (5)	C16—H16B	0.9600
C8—C9	1.399 (6)	C16—H16C	0.9600
C3—C4	1.398 (5)	C10—C11	1.378 (8)
C3—H3	0.9300	C10—H10	0.9300
C7—C6	1.379 (5)	C11—H11	0.9300
			
P3—C22—H22A	109.5	C2—C7—H7	119.3
P3—C22—H22B	109.5	P2—C17—H17A	109.5
H22A—C22—H22B	109.5	P2—C17—H17B	109.5
P3—C22—H22C	109.5	H17A—C17—H17B	109.5
H22A—C22—H22C	109.5	P2—C17—H17C	109.5
H22B—C22—H22C	109.5	H17A—C17—H17C	109.5
P3—C21—H21A	109.5	H17B—C17—H17C	109.5
P3—C21—H21B	109.5	C5—C6—C7	121.3 (4)
H21A—C21—H21B	109.5	C5—C6—H6	119.4
P3—C21—H21C	109.5	C7—C6—H6	119.4
H21A—C21—H21C	109.5	F2—C13—C12	117.9 (5)
H21B—C21—H21C	109.5	F2—C13—C8	118.5 (3)
P3—C20—H20A	109.5	C12—C13—C8	123.5 (5)
P3—C20—H20B	109.5	C5—C4—C3	121.5 (4)
H20A—C20—H20B	109.5	C5—C4—H4	119.3
P3—C20—H20C	109.5	C3—C4—H4	119.3
H20A—C20—H20C	109.5	C11—C12—C13	118.8 (5)
H20B—C20—H20C	109.5	C11—C12—H12	120.6
O—Co1—C1	39.56 (11)	C13—C12—H12	120.6
O—Co1—P3	137.86 (8)	C6—C5—C4	118.5 (4)
C1—Co1—P3	108.22 (10)	C6—C5—H5	120.8
O—Co1—P2	92.30 (8)	C4—C5—H5	120.8
C1—Co1—P2	130.17 (9)	P4—C14—H14A	109.5
P3—Co1—P2	102.91 (5)	P4—C14—H14B	109.5
O—Co1—P4	113.57 (8)	H14A—C14—H14B	109.5
C1—Co1—P4	109.76 (10)	P4—C14—H14C	109.5
P3—Co1—P4	102.18 (5)	H14A—C14—H14C	109.5
P2—Co1—P4	100.24 (5)	H14B—C14—H14C	109.5
C19—P2—C17	100.2 (2)	P2—C19—H19A	109.5
C19—P2—C18	101.8 (2)	P2—C19—H19B	109.5
C17—P2—C18	99.8 (2)	H19A—C19—H19B	109.5
C19—P2—Co1	110.71 (15)	P2—C19—H19C	109.5
C17—P2—Co1	112.49 (17)	H19A—C19—H19C	109.5
C18—P2—Co1	128.02 (16)	H19B—C19—H19C	109.5
C20—P3—C22	98.6 (3)	F1—C9—C10	116.9 (5)
C20—P3—C21	100.3 (3)	F1—C9—C8	118.9 (4)
C22—P3—C21	100.3 (3)	C10—C9—C8	124.2 (5)
C20—P3—Co1	118.24 (19)	P4—C15—H15A	109.5
C22—P3—Co1	121.41 (17)	P4—C15—H15B	109.5
C21—P3—Co1	114.39 (17)	H15A—C15—H15B	109.5
C16—P4—C15	99.3 (3)	P4—C15—H15C	109.5
C16—P4—C14	99.2 (3)	H15A—C15—H15C	109.5
C15—P4—C14	100.2 (3)	H15B—C15—H15C	109.5
C16—P4—Co1	119.15 (17)	P2—C18—H18A	109.5
C15—P4—Co1	113.11 (18)	P2—C18—H18B	109.5
C14—P4—Co1	122.00 (16)	H18A—C18—H18B	109.5
C3—C2—C7	117.0 (3)	P2—C18—H18C	109.5
C3—C2—C1	124.7 (3)	H18A—C18—H18C	109.5
C7—C2—C1	118.3 (3)	H18B—C18—H18C	109.5
O—C1—C2	116.8 (3)	P4—C16—H16A	109.5
O—C1—C8	115.1 (3)	P4—C16—H16B	109.5
C2—C1—C8	116.9 (3)	H16A—C16—H16B	109.5
O—C1—Co1	64.08 (17)	P4—C16—H16C	109.5
C2—C1—Co1	113.5 (2)	H16A—C16—H16C	109.5
C8—C1—Co1	119.9 (2)	H16B—C16—H16C	109.5
C13—C8—C9	114.2 (4)	C9—C10—C11	119.0 (6)
C13—C8—C1	125.0 (4)	C9—C10—H10	120.5
C9—C8—C1	120.7 (4)	C11—C10—H10	120.5
C2—C3—C4	120.3 (4)	C12—C11—C10	120.2 (5)
C2—C3—H3	119.8	C12—C11—H11	119.9
C4—C3—H3	119.8	C10—C11—H11	119.9
C6—C7—C2	121.3 (4)	C1—O—Co1	76.37 (18)
C6—C7—H7	119.3		
